# Feasibility of south–south collaboration in Africa: the Uganda–Mozambique perspective

**DOI:** 10.5830/CVJA-2018-030

**Published:** 2018

**Authors:** Namuyonga Judith, Lwabi Solomon, Omagino John, Yacoub Magdi, Olga Mocumbi Ana

**Affiliations:** Uganda Heart Institute Ltd, Kampala, Uganda; Uganda Heart Institute Ltd, Kampala, Uganda; Uganda Heart Institute Ltd, Kampala, Uganda; Imperial College of London, UK; Aswan Heart Centre, Cairo, Egypt; Mozambique Institute of Health Education and Research (MIHER); Instituto Nacional de Saude; Universidade Eduardo Mondlane, Maputo, Mozambique

**Keywords:** cardiovascular research, south–south partnerships, Uganda–Mozambique

Existing collaborations in health have been common between developed countries (north) and low-income countries (south). Not only have such partnerships developed capacity in African institutions, they have also enhanced skills transfer and increased research.^1^

Uganda and Mozambique are low-income countries (LICs) located in the eastern part of the African continent that have much in common. The health budgets of both countries are less than 15%, as documented in the Abuja Declaration by African heads of state and the World Health Organisation.^2^

High infant and maternal mortality rates occur in these nations, as well as chronic diseases of poverty such as rheumatic heart disease (RHD) and endomyocardial fibrosis (EMF), among others.^3^,^4^ We report here on an experience of collaboration between two institutions from these LICs using small budgets for mentorship in research and speciality training, with a focus on poverty-related cardiovascular diseases.

## Existing partnerships

The Uganda Heart Institute (UHI) is the only centre in Uganda mandated by an act of parliament to offer cardiovascular services, including diagnosis, open-heart surgery and training of doctors, nurses and other health professionals. The Institute has had some collaboration with foreign teams from the USA and UK.^5^

On the other hand, Mozambique has a heart institute that functions as a private not-for-profit organisation, and a national referral public hospital, both offering comprehensive cardiovascular services, including catheterisation laboratories and open-heart surgery, supported by collaborations with universities and hospitals from the UK, Switzerland, Portugal, France, USA and Spain.^1^ Mozambique has also prioritised research into neglected cardiovascular diseases through its National Health Institute and the Eduardo Mondlane University.

## Rationale

One of the most neglected cardiovascular diseases, EMF, is prevalent in certain areas of Mozambique and Uganda, where it is an important cause of heart failure. This restrictive cardiomyopathy usually affects underprivileged young people living in remote rural areas.

Whereas Uganda pioneered EMF-related research in the 40s,^6^ the most recent studies have emerged in Mozambique, in a rural coastal district of Inharrime, near Maputo.^4^ EMF was first described in Uganda by Davies in 1948,^6^,^7^ who reported biventricular involvement in over 50% of the autopsies and a 14% prevalence. In the 1970s, Somers and colleagues demonstrated a familial component of EMF.^8^

More recently, Mocumbi et al. used portable cardiac ultrasound to assess EMF prevalence in the general population in Inharrime, which was found at 18.9%.^3^ Diagnostic criteria are currently based on echocardiography,^4^ which increases sensitivity and is becoming increasingly accessible in the affected countries.

The aetiology of EMF has been linked to genetic and environmental factors,^4^,^9^ but the exact cause of the disease remains unknown. Clinical features depend on the severity of the disease^10^ and how affected the ventricles are, and characteristically include prominent ascites disproportionate to little or no lower-limb oedema. Death usually results from heart failure, thromboembolism and arrhythmias.^11^

Among individuals with advanced disease, surgery has shown substantial benefits,^12^ but remains challenging and palliative, as it improves symptoms only, carrying high morbidity and mortality rates.^13^ Therefore, visiting teams to endemic areas are not keen to operate on such cases.

## Mozambique–Uganda collaboration: feasibility study on 10-year follow up of EMF

While cases of EMF in Uganda seem to have reduced over the years, the reverse is true in Mozambique. A comprehensive programme has been started by Mozambique to understand the epidemiology, clinical characterisation and natural history of the disease, and to explore new therapeutic options.

Teams of the UHI and NPHI (National Public Health Institute) decided to exchange knowledge by starting a collaborative project with both training and research arms. A paediatric cardiologist (Judith Namuyonga) from the UHI Paediatric Cardiology Department, mentored by Peter Lwabi, had a placement arranged in Mozambique’s NPHI, under the mentorship of the local principal investigator and paediatric cardiologist (AO Mocumbi), to participate in the feasibility study with a 10-year follow up. The presence of this Ugandan colleague was particularly important to strengthen the capacity of the Mozambican team to perform cardiac ultrasound in the field, a crucial part of the follow-up study that can only be performed by the 16 existing cardiologists in Mozambique.

This exchange occurred between 5 and 16 September 2017 in Maputo city and in the rural district of Inharrime, located 400 km from Maputo. The study was co-funded by the NPHI and UHI, as well as the Aswan Heart Centre and Chain of Hope, where EMF surgery will potentially be done for selected patients, owing to the presence of Prof Magdi Yacoub, who has in-depth experience on the surgical approach to the disease and mentored Mocumbi in her post-graduate studies.^12^,^14^ This personnel link has now been used to promote exchange of researchers between Mozambique, Uganda and Egypt, through projects focusing on the epidemiology and management of EMF.

Research mentorship consisted of skills transfer in grant application processes, including acquisition of unique Data Universal Number System (DUNS) number, data management, budgeting and use of electronic platforms for data entry. The use of research administration number (eRA Commons D) was also introduced to Namuyonga.

The mentor (Mocumbi) and the mentee/fellow (Namuyonga) travelled from Maputo to Inharrime where the team held several preparatory meetings with the village local leaders and did house-to-house visits. Portuguese is the national language and was the most frequently used medium of communication, which was a limitation to Namuyonga during the family visits. Therefore she was in charge of performing cardiac ultrasound while Mocumbi consulted participants, collected clinical history and took verbal autopsy, whenever applicable.

Using GPS, we were able to find all houses previously visited, and have obtained consent from all heads of households. In this early study phase, we visited 25 locations from four administrative areas, where we performed 31 cardiac ultrasounds.

Extreme poverty was noted in this geographic area, coupled with the dry tropical weather. Few gardens of cassava were seen, most people could only afford a single meal a day, and there was low access to clean water.

## Immediate outcomes

This report demonstrates the feasibility of collaboration between LICs in sub-Saharan Africa, including clinical research mentorship, the conduction of high-quality, patient-orientated research, and community-based research. Immediate outcomes were the reinforcement of Mozambique’s capacity for performing field ultrasound, creation of a network of three African countries, and the initiation of other projects on cardiovascular risk factors in young populations.

As African trainees do not easily get hands-on training in affluent/well-developed nations, this kind of collaboration could be one of the avenues to overcome this problem in certain areas of clinical and research training. South-to-south research collaboration projects may be a platform to foster these partnerships and promote efficient use of resources in underserved areas.
